# Increase in Docetaxel-Resistance of Ovarian Carcinoma-Derived RMG-1 Cells with Enhanced Expression of Lewis Y Antigen

**DOI:** 10.3390/ijms12117323

**Published:** 2011-10-26

**Authors:** Fan Zhang, Juanjuan Liu, Bei Lin, Qing Liu, Yue Zhao, Liancheng Zhu, Yingying Hao, Shulan Zhang, Masao Iwamori

**Affiliations:** 1 Department of Obstetrics and Gynecology, Shengjing Hospital Affiliated to China Medical University, Shenyang 110004, Liaoning, China; E-Mails: converting@163.com (F.Z.); converting@sina.com (J.L.); liuq88@163.com (Q.L.); zhuzhu008@sina.com (L.Z.); yingyinghao@126.com (Y.H.); zhangshl@126.com (S.Z.); 2 Department of Obsterics and Gynecology, Beijing Haidian Maternal and Child Health Hospital, Beijing 100080, China; 3 Department of Obstetrics and Gynecology, Second Hospital Affiliated to Dalian Medical University, Dalian 116023, Liaoning, China; E-Mail: zhaoyy@sina.com; 4 Department of Biochemistry, Faculty of Science and Technology, Kinki University, 3-4-1 Kowakae, Higashiosaka, Osaka 577-8502, Japan; E-Mail: iwamori@163.com

**Keywords:** ovarian cancer, Lewis Y antigen, docetaxel, drug resistance

## Abstract

Epithelial carcinomas of the ovary exhibit the highest mortality rate among gynecologic malignancies. Studies found that the metabolism of glycolipids or carbohydrates is associated with acquirement of anticancer drug-resistance by cancer cells. This study was to characterize possible involvement of Lewis Y (Le^Y^) antigen in the drug-resistance of cancer cells. We transfected the α1,2-fucosyltransferase gene into human ovarian carcinoma-derived RMG-1 cells and established RMG-1-hFUT cells with enhanced expression of Le^Y^. We determined the effects of docetaxel on the survival of cells by MTT assaying and observed the apoptosis of cells in the presence of docetaxel by flow cytometric analysis and by transmission electron microscopy. Plasma membranes and intracellular granules in RMG-1-hFUT cells were stained with anti-Le^Y^ antibody, the intensity of the staining was higher than that in control cells. The RMG-1-hFUT cells exhibited higher resistance to docetaxel than the control cells with regard to the docetaxel concentration and time course. After treatment with 10 μg/mL docetaxel for 72 h, the control cells, but not RMG-1-hFUT, contained abundant positively stained cell debris due to disintegration of the cytoskeleton. On transmission electron microscopy, although the control cells treated with docetaxel as above showed the following morphology, *i.e.*, absence of villi, cells shrunken in size, pyknosis, agglutinated chromatin and cell buds containing nuclei in the process of apoptosis, the RMG-1-hFUT cells showed only agglutinated chromatin and vacuoles in the cytoplasm. In summary, cells with enhanced expression of Le^Y^ were shown to acquire docetaxel-resistance, indicating the possible involvement of glycoconjugates in the drug-resistance.

## 1. Introduction

Epithelial carcinomas of the ovary exhibit the highest mortality rate among gynecologic malignancies, that is, a 5-year survival rate in patients treated with cytoreductive surgery, and combined with chemotherapy only 30–40%. Since the main reason for the failure of chemotherapy in the patients is the drug resistance of cancer cells, clarification of the mechanism underlying the resistance is required to improve the systemic treatments including chemotherapy for patients. The chemoresistance mediated by ATP-binding cassette (ABC) transporters, such as multidrug resistance (MDR) or multidrug resistance-associated protein (MRP) transporters, is mainly due to a decrease in the cellular accumulation of anticancer drugs due to extrusion of the drugs out of the cells through the transporters [1,2]. While, the resistance associated with glucosylation of ceramides is related to the removal of virulent ceramides, which are generated from sphingomyelin on activation of sphingomyelinase in the anticancer drug-induced apoptotic pathway [3,4]. Also, enhanced expression of LacCer and Gb_3_Cer with 2-hydroxy fatty acids and longer carbohydrate chains, together with that of transporters, was characteristically observed in paclitaxel- and cisplatin-resistant ovarian carcinoma-derived cells [5], and GM3 and Le^b^ were found in paclitaxel-resistant cells [6], indicating that the metabolism of glycolipids or carbohydrates is associated with acquirement of anticancer drug-resistance by cancer cells. More directly, we previously demonstrated that transfection of the α1,2-fucosyltransferase gene into ovarian carcinoma-derived RMG-1 cells resulted in an increased amount of Le^Y^ antigen and enhanced anticancer drug resistance against 5-fluorouracil (5-FU) and carboplatin [7,8]. In fact, Lewis-related carbohydrate-structures were reported to be transformation-related antigens, that is, sialyl Le^a^ for the metastatic potential [9,10], and Le^b^, Le^Y^ and H antigens for the grades of dysplasia and malignancy [11,12]. To further characterize the relationship between Lewis antigens and chemotherapeutic resistance of ovarian carcinoma-derived cells, we prepared RMG-1 cells exhibiting enhanced expression of Le^Y^ antigen by transfection with the FUT-1 gene and compared their cell biological properties including resistance against docetaxel, which is a semi-synthetic taxol derivative whose cytotoxic effect involves promotion polymerization of tubulin and inhibition of the depolymerization of microtubules for three times longer than in the case of paclitaxel.

## 2. Results

### 2.1. Expression of Le^Y^ Antigen in Cells Transfected with the hFUT Gene

**Figure 1:**
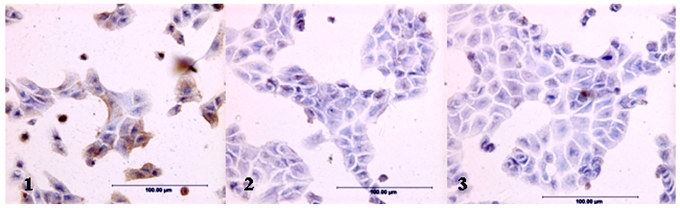
Immunocytochemical staining of cells with anti-LeY antibodies: (**1**) RMG-1-hFUT; (**2**) RMG-1; and (**3**) RMG-1(-).

On immunocytochemical staining with anti-Le^Y^ antibody, RMG-1-hFUT cells established by transfection with the hFUT gene were found to express Le^Y^ antigen at a significantly higher level than the original RMG-1 cells and the RMG-1(-) cells transfected with the vector alone, and the Le^Y^-antigen in the RMG-1-hFUT cells were distributed in both the plasma membrane and intracellular granules (Figure 1).

### 2.2. Effects of Docetaxel on the Survival Rates of Cells

**Figure 2:**
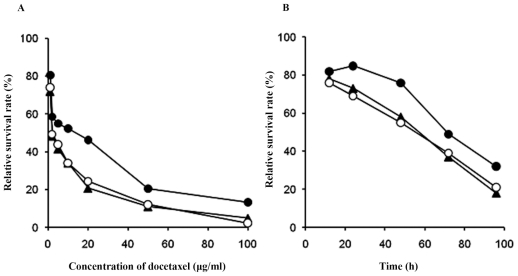
Relative survival rates of cells cultured in the presence of docetaxel. ○**–**○, RMG-1; ▴**–**utrif, RMG-1(-);●**–**●, RMG-1-hFUT. (**A**) cells were cultured in medium containing different concentrations of docetaxel for 72 h; (**B**) cells were cultured in medium containing 10 μg/mL docetaxel for various times. Viable cells were determined by MTT assaying and the relative survival rates were calculated in comparison to those of cells cultured without docetaxel.

The relative survival rates of RMG-1, RMG-1(-) and RMG-1-hFUT cells cultured in the presence of docetaxel to those cultured without docetaxel were compared as functions of the docetaxel concentration and time course. As shown in Figure 2, RMG-1-hFUT cells were more resistant against docetaxel than RMG-1 and RMG-1(-) cells, and the concentrations of docetaxel giving the half survival rates (IC_50_) were as follows, 7.98 ± 1.31 μg/mL for RMG-1-hFUT, 4.76 ± 1.42 μg/mL for RMG-1 and 4.60 ± 1.18 μg/mL for RMG-1(-). Thus, the concentration of docetaxel at IC_50_ for RMG-1-hFUT cells was 1.7 times higher than those for RMG-1 and RMG-1(-) cells. Based on three separate experiments, the statistical significance of the docetaxel-resistance of RMG-1-hFUT cells as compared to that of RMG-1 and RMG-1(-) cells was evident, *i.e.*, *P* < 0.05 and *t* = 2.88 and 3.34, respectively (Table 1).

### 2.3. Docetaxel-Induced Apoptosis

**Figure 3:**
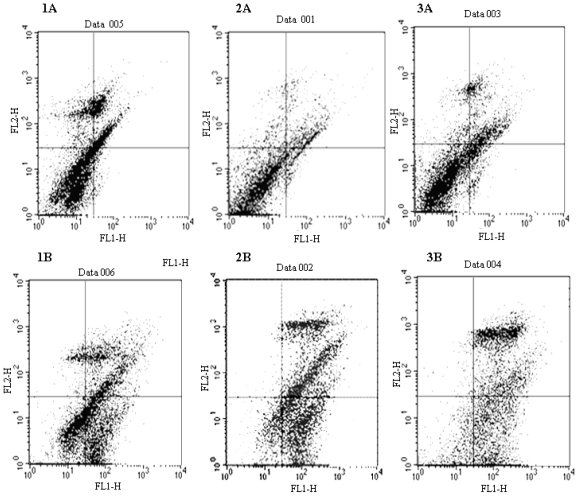
Flow cytometric analysis of cells after treatment with and without docetaxel. Cells cultured without (**A**) and with (**B**) docetaxel (10 μg/mL) for 72 h were stained with annexin-V-FITC/PI according to the manufacturer’s instructions and then analyzed with a FACS Calibur. (**1**) RMG-1-hFUT; (**2**) RMG-1; and (**3**) RMG-1(-).

**Figure 4:**
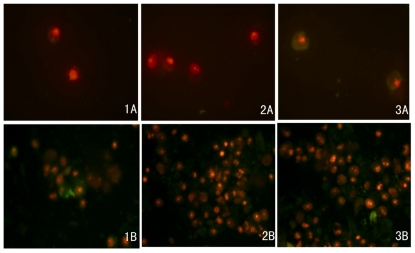
Immunofluorescence microscopy of cells stained with annexin-V- FICT/PI after treatment with and without docetaxel. Cells cultured without (**A**) and with (**B**) docetaxel (10 μg/mL) for 72 h were stained with annexin-V- FICT/PI and then examined under a fluorescence microscope (× 400). (**1**) RMG-1-hFUT; (**2**) RMG-1; and (**3**) RMG-1(-).

The frequency of apoptosis on cultivation of cells in the presence of docetaxel was determined by staining with annexin-V-FITC/PI, followed by flow cytometric analysis. As shown in Figure 3 and Table 2, RMG-1-hFUT cells after treatment with docetaxel at the concentration of 10 μg/mL for 72 h exhibited a significantly lower proportion of apoptotic cells than RMG-1 and RMG-1(-) cells (*P* < 0.01), *i.e.*, 65.3% for RMG-1-hFUT, 83.7% for RMG-1(-), and 82.2% for RMG-1. No significant difference was observed between those of RMG-1(-) and RMG-1 (*P* > 0.05). Then, cells stained with annexin-V-FITC/PI were examined under a fluorescence microscope. As shown in Figure 4, RMG-1 and RMG-1(-) cells, in comparison to RMG-1-hFUT ones, were intensively stained and became smaller in size, and exhibited apoptosis with disintegration of the cytoskeleton generating cell debris, which exposed phosphatidyl serine, which is reactive with annexin V. The results clearly indicated that RMG-1-hFUT cells were more resistant against docetaxel than RMG-1 and RMG-1(-) cells.

### 2.4. Ultrastructure of Cells Examined by TEM

**Figure 5:**
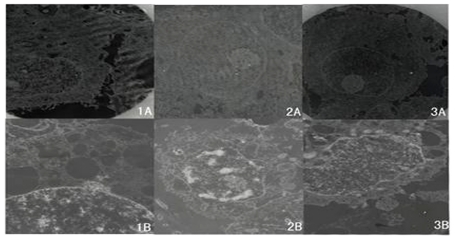
TEM of cells after treatment with and without docetaxel. Cells cultured without (**A**) and with (**B**) docetaxel (10 μg/mL) for 72 h were examined by TEM. (**1**) RMG-1-hFUT; (**2**) RMG-1; and (**3**) RMG-1(-).

Cells not treated with docetaxel exhibited a similar morphology to cancer cells, *i.e.*, irregular shaped cells, abundant microvilli on cell surfaces, large number of intracellular structures in the cytoplasm, obvious nucleolus, uniform chromatin, and a rich cytoplasm (Figure 5). RMG-1 and RMG-1(-) cells cultured in the presence of docetaxel showed the absence of villi, a decrease in size, pyknosis, agglutinated chromatin, and cell buds containing cell nuclei, and organs protruded out of the cell surface, and apoptotic bodies were finally formed, these being the typical morphological characteristics of cell apoptosis. In contrast, RMG-1-hFUT cells showed only agglutinated chromatin and vacuoles in the cytoplasm, *i.e.*, no apoptotic bodies, indicating suppression of the process of apoptosis by docetaxel.

## 3. Discussion

Cell surface glycoconjugates are frequently altered in association with transformation and are implicated in the malignancy of cancer cells, including their potential for metastasis and anticancer drug-resistance. As to ovarian carcinomas, Le^Y^ oligosaccharide, Fucα1-2Galβ1-4GlcNAc(3-1αFuc)-, which belongs to the lacto-series type 2 chain family, has been reported to be expressed in 60–90% of patients as a cancer-associated antigen, and its amount in cancer cells to be closely related with a poor prognosis [13]. We also observed that the quantity of Le^Y^ in primary ovarian carcinomas was similar to that in metastatic ones, and the more serious the malignancy, the higher the quantity of Le^Y^ antigen [7], as reported by others [14]. Also, an ovarian carcinoma-associated antigen, CA125, which has been widely applied for clinical diagnosis, contained the Le^Y^-structure in its molecule [15], and accordingly an approach involving immunotherapy toward Le^Y^ as the target antigen was examined as a better therapeutic procedure, since conventional surgery and chemotherapy do not significantly increase the survival rate of ovarian cancer patients [16].

In our previous study [7], we established mutant cell lines of ovarian carcinoma-derived RMG-1 cells by transfection of the α1,2-fucosyltransferase gene, and the resultant cells were found to contain a 20 times higher amount of Le^Y^-glycolipids than the original cells, and simultaneously to exhibit more malignant behavior, such as chemotherapeutic drug-resistance toward 5-FU and an increased metastatic potential compared to the original cells. The involvement of α1,2-fucosyltransferase in drug-resistance toward 5-FU has also been reported in mice treated with 5-FU and human colon carcinoma-derived cells with 5-FU-resistance, but H-2 and Le^b^ antigens were utilized as the drug-resistance-related glycoconjugates synthesized by α1,2-fucosyltransferase [17]. Since Le^x^-glycolipids were contained in RMG-1 cells in a significantly higher amount, the major glycolipid synthesized on activation of α1,2-fucosyltransferase in RMG-1 cells was expected to be Le^Y^-glycolipid [7], and also the same Le^Y^-structure should be expressed on glycoproteins. Consequently, we attempted to establish RMG-1 cells with enhanced expression of Le^Y^ antigen at a higher level than previously attained by selection of Le^Y^-positive cells with a flow cytometer, and successfully established RMG-1-hFUT cells exhibiting enhanced expression of Le^Y^.

On comparing the cellular behavior after treatment with docetaxel of RMG-1-hFUT cells with that of control cells, RMG-1-hFUT cells were found to exhibit stronger drug-resistance than control cells. RMG-1-hFUT cells exposed to 10 μg/mL docetaxel for 72 h showed only agglutinated chromatin and vacuoles in the cytoplasm, *i.e.*, they did not produce apoptotic bodies, on analysis by TEM, whereas the progress of apoptosis was evidently observed in the control cells treated under the same conditions. Also, reduced apoptosis of RMG-1-hFUT cells treated with docetaxel, in comparison to that of control cells, was observed on staining with annexin-V-FITC/PI, followed by fluorescence microscopy and flow cytometry. Thus, enhanced expression of Le^Y^ on transfection of the α1,2-fucosyltransferase gene was associated with the drug-resistance against docetaxel and also that against carboplatin and 5-FU, providing a molecular marker of the drug-resistance.

The mechanism of Le^Y^ antigen’s effect on drug resistance needs further research. Modification of the activity of epidermal growth factor receptor (EGFR), which is the glycoprotein with Le^Y^ in the carbohydrate structure [18], was thought to be one of the mechanisms. EGFR is a tyrosine kinase, which is highly expressed in ovarian cancers, and activates the PI3K-MAPK pathway leading to protein kinase-B (PKB) and Bcl-2-phosphorylation. The EGFR-mediated signal transduction pathway was revealed to be inhibited by anti-Le^Y^ antibodies, which leads to apoptosis of cancer cells [19]. In this connection, inhibitors of PI3K and JAK2 inhibit Le^Y^ and H-mediated endothelial cell formation in the process of angiogenesis, indicating the involvement of Le^Y^ and the H-structure in the growth factor-mediated signal transduction pathway [14]. We obtained proof of the high MRP-1 promoter activity observed in the presence of EGF in the transfection assays, supported an effect at the transcription level [20]. According to the reports, the PI3K pathway known to release free E2 factor (E2F) [21], and the results of Lee H’s research showed that E2F is the activator of Topo I mRNA’s transcription [22]. CD44 is another cytomembrane protein with Fucα1-2Galβ1-4GlcNAc(3-1αFuc)-, which play an important role in cell adhesion and signal transduction. Multidrug resistance protein-2 (MRP-2) expression was induced in H322/CD44s cells cultured on hyaluronan. It also resulted in the transcription of MDR-1 and the expression of P-glycoprotein on lymphocytes in a dose-dependent manner. Research showed CD44 correlation with the expression of PKC-α [23]. Wang [24] found that all cell signal pathways were activated including increased expression or phosphorylation of PKB and PKN in α1,3-FucT-VII overexpressing human H7721 hepatocarcinoma cell line by transfecting the cDNA of α1,3-fucosyltransferases-VII (α1,3-FucT-VII) into the cell line. The same discovery showed up in our experiments by oligonucleotide microarray. Compared with RMG-1, there were 88 genes with different expressions in RMG-1-hFUT. After investigating the different expressed genes, we found RMG-1-hFUT cells showed obvious alterations in protein binding, nucleotide binding, cell proliferation, DNA-dependent regulation of transcription, signal transduction, protein amino acid phosphorylation, transcription, cell adhesion [25]. We presume that the changes of content and composing component of glycan chain on cell surface occurred after we transfected the cDNA of FUT1 which possibly induced or up-regulated the expression of resistance-related protein genes by several signal transduction pathways. Our experiments displayed that Le^Y^ antigen could improve the resistance of carcinoma cells to chemotherapeutics by boosting the corpuscular activity. So the drug resistance of ovarian carcinoma-derived RMG-1 cells with enhanced expression of Le^Y^ antigen is the result of multidrug resistant mechanisms combined action.

## 4. Materials and Methods

### 4.1. Materials

The following reagents were purchased from commercial sources: expression vector pcDNA3.1(-) and a TA cloning kit from Invitrogen (San Diego, CA, USA), *E. coli* (competent cells) JM109 from Toyobo (Tokyo, Japan), restriction endonucleases, BamHI, EcoRI, and G418 (geneticin) from Gibco, cell transfection and NucleoBond plasmid kits from GE Healthcare (Piscataway, NJ, USA), AmpliTaq Gold^TM^ and a Bigdye^TM^ terminator cycle sequencing ready reaction kit from Perkin-Elmer/Applied Biosystems (Foster City, CA, USA), an immunocytochemical SABC kit from Boshide Biotech Co (Wuhan, China), a mouse monoclonal anti-Le^Y^ antibody from Santa Cruz (CA, USA), docetaxel from Shandong Qilu Pharmaceutical Co. Ltd (China), Dulbecco’s modified Eagle’s medium (DMEM) and fetal bovine serum (FBS) from Hyclone (Logan, UT, USA), trypsin, ethylenediamine tetraacetic acid (EDTA), and 3(4,5-dimethyl-2-thiazolyl)-2,5-diphenyl tetrazolium (MTT) from Amresco (Solon, OH, USA), and an annexin-V-FITC/PI kit from Jingmei Biotech Co., Ltd. (Shenzhen, China).

### 4.2. Cell Culture

Human ovarian carcinoma (clear cell type)-derived RMG-1 cells and their transfectants, RMG-1(-) and RMG-1- hFUT cells, were cultured in DMEM containing 10% FBS, 100 U/mL penicillin and 0.1 mg/mL streptomycin in a humidified incubator at 37 °C under a 5% CO_2_ atmosphere.

### 4.3. Transfection of the Fucosyltransferase Gene

The human α1,2-fucosyltransferase gene (FUT-1) was amplified by PCR with human leukocyte genomic DNA as a template and primers according to the human FUT-1 gene sequence (GenBank Accession Number: M35531), sense primer, 5′-CATGTGGCTCCGGAGCCATCGTC-3′, and antisense primer, 5′-GCTCTCAAGGCTTAGCCAATGTCC-3′, under the following conditions: denaturation at 94 °C for 9 min, followed by 25 cycles of 94 °C, 1 min, 65 °C, 1.5 min, and 72 °C, 2 min, and then extension at 72 °C for 10 min. The PCR products were ligated into the PCR 2.1 vector to clone FUT-1 gene, and its DNA sequence was determined by means of the dideoxynucleotide chain-termination method with the BigDye terminator cycle sequenceing ready reaction kit and a DNA sequencer (ABI Genetic Analyzer; Perkin-Elmer/Applied Biosystems). Then the FUT-1 gene in pCR2.1 was cut out by digestion with restriction enzymes, BamHI and EcoRI, and ligated into the BamHI and EcoRI sites of the pcDNA3.1 vector (pcDNA3.1-hFUT). pcDNA3.1-hFUT and the vector alone were transfected into RMG-1 cells with a vector transfection kit, according to the instructions for the kit to establish RMG-1-hFUT and RMG-1(-) cells, respectively. The resultant transfectants were initially selected by cultivation with medium containing an aminoglycoside antibiotic, G418, at 400 μg/mL concentration, and were maintained at 200 μg/mL for 15 days. Finally, RMG-1-hFUT cells exhibiting significantly higher expression of Le^Y^-structure than that of the original RMG-1 and the RMG-1(-) cells were selected by flow cytometry using a FACS Calibur, with monoclonal anti-Le^Y^ antibodies.

### 4.4. Immunocytochemical Assaying

RMG-1, RMG-1(-) and RMG-1-hFUT cells cultured in chamber slides were fixed with 4% paraformaldehyde and then stained according to the instructions for the SABC kit. The primary antibody, mouse monoclonal anti-Le^Y^ antibody, biotin-labeled goat monoclonal anti-mouse IgG antibody and alkaline phosphatase-labeled avidin were used at 1:100 dilution, and specimens were observed under a light microscope (IX71, Olympus, Tokyo, Japan).

### 4.5. Cell Viability Determined by MTT Assaying

Cell suspensions (3 × 10^3^ cells/200 μL) were inoculated into the wells of a 96-well plate and cultured for 20 h, followed by cultivation in the medium containing docetaxel at concentrations of 1, 2, 5, 10, 20, 50 and 100 μg/mL for 72 h, and also in the medium containing 10 μg/mL docetaxel for different periods, 12 h, 24 h, 48 h, 72 h and 96 h. Then, 20 μL of MTT (5 mg/mL) was added and the plate was incubated for 4 h, and the resultant formazan in the viable cells was dissolved with 150 μL of dimethyl sulfoxide with a horizontal oscillator until visible purple crystalline matter was dissolved completely. The optical density of each well was determined at 570 nm with a ELISA monitor (Sunrise RC TS TC, Tecan). The assays were repeated three times, and the concentrations of docetaxel giving the IC_50_ were determined.

### 4.6. Flow Cytometry

To compare the ratios of apoptosis and necrosis for RMG-1, RMG-1(-) and RMG-1-hFUT cells after treatment with docetaxel, cells cultured in the medium containing 10 μg/mL docetaxel for 72 h were stained with the annexin-V-FITC/PI kit according to the instructions, and then analyzed by flow cytometry (FACS Calibur) and fluorescence microscopy (IX71, Olympus).

### 4.7. Transmission Electron Microscopy (TEM)

Cells (1 × 10^6^ cells) cultured in the medium containing 10 μg/mL docetaxel for 72 h were fixed with 2.5% glutaraldehyde for 2 h. After washing the cells with phosphate-buffered saline (PBS), sections for TEM were prepared as follows, fixation with 1% osmium tetroxide at 4 °C for 30 min, dehydration with several concentrations of acetone, embedding with Epon812, preparation of ultra thin slices, and staining with uranyl acetate and lead acetate, followed by examination at × 6000 by TEM (JEM-1200EX, JEOL, Tokyo, Japan).

## 5. Conclusions

In summary, cells with enhanced expression of Le^Y^ were shown to acquire docetaxel-resistance, indicating the possible involvement of glycoconjugates in drug-resistance.

## Figures and Tables

**Table 1:** Concentrations of docetaxel giving 50% survival rates (IC_50_) as determined by MTT assaying. Data are based on three separate experiments and the relative docetaxel-resistance of RMG-1-hFUT cells was compared to that of RMG-1 (a) and RMG-1(-) (b) cells. Please check the highlighted cells.

Cell line	IC_50_ (μg/mL)	*t*^a^	*P*^a^	*t*^b^	*P*^b^
RMG-1-hFUT	7.98 ± 1.31	2.88	0.05	3.34	0.03
RMG-1(-)	4.60 ± 1.18	0.16	0.88		
RMG-1	4.76 ± 1.42				

**Table 2:** Apoptotic cells after treatment with 10 μg/mL docetaxel, as analyzed by flow cytometry after staining of the cells with annexin-V-FITC/PI. Data are based on three separate experiments and the proportion of apoptotic cells for RMG-1-hFUT cells was compared to those of RMG-1 (a) and RMG-1(-) (b) cells. Please check the highlighted cells.

Cell line	Apoptotic cells (%)	*t*^a^	*P*^a^	*t*^b^	*P*^b^
RMG-1-hFUT	65.3 ± 2.1	7.42	0.006	9.50	0.002
RMG-1(-)	83.7 ± 5.5	0.14	0.71		
RMG-1	82.2 ± 6.4				
